# Is Current Research on How Climate Change Impacts Global Food Security Really Objective?

**DOI:** 10.3390/foods10102342

**Published:** 2021-09-30

**Authors:** Wangang Liu, Yiping Chen, Xinhua He, Ping Mao, Hanwen Tian

**Affiliations:** 1 SKLLQG, Institute of Earth Environment, Chinese Academy of Sciences, Xi’an 710075, China; liuwangang@ieecas.cn (W.L.); tianhanwen2144882@163.com (H.T.); 2 College of Resource, Southwest University, Chongqing 610041, China; xinhua.he@uwa.edu.au; 3 Chengdu Institute of Biology, Chinese Academy of Sciences, Chengdu 610041, China; maoping@cib.ac.cn

**Keywords:** climate change, food security, global, regions, facility

## Abstract

Global food insecurity is becoming more severe under the threat of rising global carbon dioxide concentrations, increasing population, and shrinking farmlands and their degeneration. We acquired the ISI Web of Science platform for over 31 years (1988–2018) to review the research on how climate change impacts global food security, and then performed cluster analysis and research hotspot analysis with VosViewer software. We found there were two drawbacks that exist in the current research. Firstly, current field research data were defective because they were collected from various facilities and were hard to integrate. The other drawback is the representativeness of field research site selection as most studies were carried out in developed countries and very few in developing countries. Therefore, more attention should be paid to developing countries, especially some African and Asian countries. At the same time, new modified mathematical models should be utilized to process and integrate the data from various facilities and regions. Finally, we suggested that governments and organizations across the world should be united to wrestle with the impact of climate change on food security.

## 1. Introduction

The Food and Agriculture Organization (FAO) of the United Nations Organization (UNO) has defined food security as “when all people have physical and economic access to sufficient, safe and nutritious food to meet their dietary needs and food preferences for an active and healthy life at all time” [1].

Based on this definition, the current global food security situation cannot be considered to be optimistic. The world population will increase to 9.1 billion in 2050 [1] whereas a 36% or even higher increase in crop production (about 1.0 billion tons) is needed to maintain the global average food consumption at the current level [2]. However, the global cultivated farmland area has been continuously declining from 0.415 hectares (ha) per capita in 1961 to 0.207 ha in 2017 because of a rapidly increasing population, urbanization, and farmland degeneration globally [3,4,5]. Worse still, although the proportion of the world’s undernourished population has declined from an estimated 980 million in 1990–1992 to about 850 million in 2010–2012, nearly 30% of the world’s population (approximately 2 billion people out of the global population of over 7 billion) should be considered to be in the “food insecure” status in the strict sense because they fall short in one or more of FAO’s dimensions of food security [6,7]. The world’s population has increased by 60% whereas crop yields are reaching a plateau. In recent years, a few crises have occurred, notably in 2007–2008 and 2010–2011, which have affected food security worldwide [8].

Climate change also threatens food security [9,10]. The atmospheric carbon dioxide (CO_2_) concentration has been rising from 280 ppm in the Industrial Revolution era to 408 ppm in 2018 and is predicted to increase to 936 ppm in 2100 [11,12]. The enhancement of CO_2_ concentration has resulted in a 0.76 °C increase in global surface temperature since the Industrial Revolution, with a 0.13 °C of decadal increment since 1950 [4]. Such a rapid CO_2_ increase will lead to a 1.4 °C–5.8 °C increase in the global surface temperature in 2100 [5,9]. The elevated CO_2_ concentration could affect food security directly via changes in air temperature, precipitation, pest attacks, disease outbreaks, and so on [10,11,13,14]. A previous study estimated that the yield of maize, rice, and wheat has declined between 2.5% and 3.8% globally in the last three decades because of climate change, and most of this decline has occurred in arid and semi-arid regions [15]. Some others have forecasted that the negative impacts of climate change on crop productivity would be more severe under more intense warming scenarios, with a median yield loss near 15% in the most intense warming scenarios [16]. Moreover, the quality of agricultural products, for example, protein content, would likely decrease and susceptibility to insect pests increase under increased CO_2_ fertilization [17,18]. As a result, against the background of global warming, the derived precipitation changes, pest and disease outbreaks, and increasing population would aggravate global food insecurity [7,15,19].

**Figure 1:**
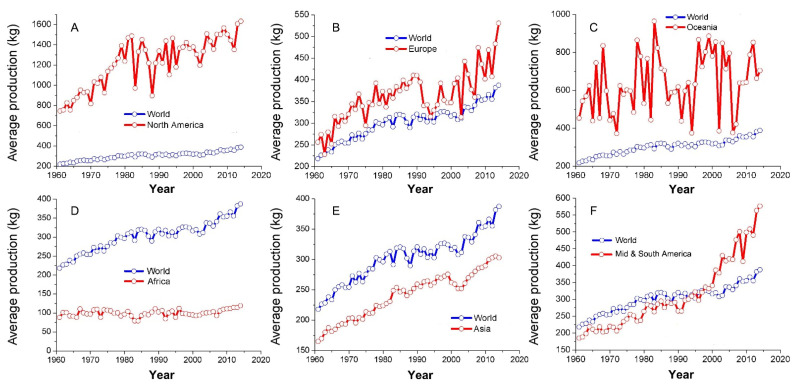
The world average grain production change in different continents ((**A**) North America, (**B**) Europe, (**C**) Oceania, (**D**) Africa, (**E**) Asia, and (**F**) Mid and South America) from 1960 to 2014.

**Figure 2:**
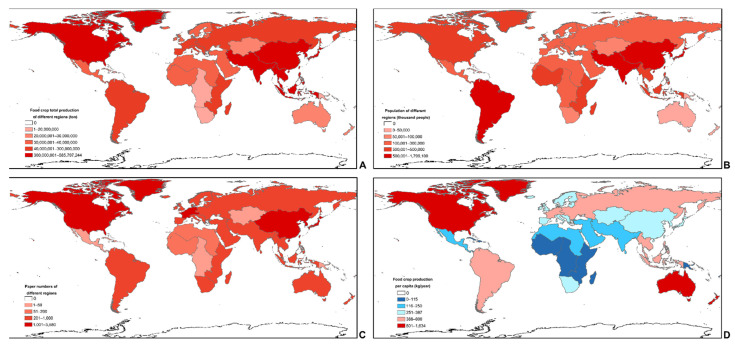
Comparisons on food crop total production (**A**), population (**B**), paper numbers (**C**) and food crop production per capita (**D**) at different continents. The figure (**A**–**D**) data were collected in 2014, while figure (**C**) was collected from 1900 to 2018. In figure (**D**), the regions in which production per capita was below the world average in 2014 (387 kg) were colored in blue and the one above world average in red.

At the intercontinental level, with a total population of 5 billion (75% of the world’s population), Asia and Africa were the only two continents where per capita food production was lower than the world average (FAO statistical database; Figure 1 and Figure 2D). Based on medium-variant projection, the Asian population in 2050 will be under control or even decline whereas the population of Africa is very likely to increase, perhaps approach, or even exceed the population of Asia [20,21]. Food security is therefore under growing threat because of low modernization of agriculture, inappropriate cropping systems, and a large population in many countries of these two continents [22]. In addition, under the potential threat of climate change and regional wars, African food security is in a perilous condition [23]. For example, in some regions of northern, eastern, and southern Africa, the average temperature increased over 1 °C between 1970 and 2000 [24].

To offset the ongoing climate change threat, researchers have made significant efforts on this topic. However, as mentioned above, several studies had shown that different regions faced different climate change intensities and were under different potential climate change threats [25,26]. So, we want to know if current studies have paid enough attention to the regions under high climate change threat. If not, how big the gaps were among research levels of different regions? Therefore, by using the ISI Web of Science Core Collection platform of Clarivate Analytics Company (Philadelphia, PA, USA; http://apps.webofknowledge.com/ (accessed on 29 September 2021)), our research depicted the current research position of the influences of climate change on food security. By analyzing and discussing the retrieval results, this study attempted to reveal the questions mentioned above.

## 2. Materials and Methods

### 2.1. Climate Change Influences on Food Security Data

On the ISI Web of Science Core Collection platform, the retrieval period was from 1900 to 2018 and the retrieval topic was “TOPIC = ((“climate change”) and ((cereal or crop or grain or maize or wheat or rice or bean or sorghum or barley) near (yield or production))) OR (((“climate change”) and “food security”)) OR ((“climate change”) and ((cereal or crop or grain or maize or wheat or rice or bean or sorghum or barley) near quality)).” The keywords, published years, institutes, researchers, research regions, and number of times the publications were cited were extracted for further study (all results in Supplement file S1 and S2).

Based on the extracted keywords, keyword cluster analysis and research hotspot analysis were performed with VosViewer [27]. The cluster analysis was based on similarities and connections between the keywords from different research papers indexed in the ISI Web of Science database. The research hotspot analysis was based on the occurrence frequency of keywords from the search results. The values of number of published papers, population, crop production, and per capita food production in different regions were generated in Arc GIS 10.2 (ESRI, West Redlands, CA, USA) and modified with Adobe Photoshop CS 6 software (San Jose, CA, USA).

### 2.2. Global and Regional Food Crop Production Data

The global and regional food crop production data were collected from the FAO statistical database (http://www.fao.org/faostat/en/#data (accessed on 29 September 2021)) from 1961 to 2014 (all results in Supplement file S3). After sorting the data using Microsoft Excel, charts were generated with Origin 8 software. Exceptionally, the data of Central Asia were only available from 1992 to 2014 because of lacking historical records. The figures of the relationships among policy suggestion, experiment equipment, model research, and potential social problems were drawn and modified with Adobe Photoshop CS 6 software.

## 3. Results

### 3.1. Global and Regional Food Crop Production

Based on crop production data downloaded from FAO statistical database, the per capita food production largely varies across continents (Figure 1). Given advanced technology and equipment, the per capita food production of North America is much higher and had been about four times higher than the world average in the past decades. Similarly, the per capita food production of Europe was also good, which was higher than the world average in the past decades. Although higher than the world average, the per capita food production of Oceania fluctuated widely in the past decades. In poor harvest years especially, the per capita food production of Oceania was close to the world average. Lower than the world average before the mid-1990s, the per capita food production of Mid and South America has been increasing rapidly and exceeding the world average since then. At present, it is approaching the European average. Different from that of the continents mentioned above, the per capita food production of Asia was slightly lower than the world average and the rising trends were also similar in the past decades. Corresponding to the worst food security situation in the world, the per capita food production of Africa was far below the world average. In contrast to the rising world average, the per capita food production of Africa did not rise noticeably in the past several decades. According to the previous climate change prediction model, these regions were under high climate change risk [28]. Basing on the results, most countries in Asia and Africa were under high food insecurity risk. Therefore, more attention should be paid to these regions.

### 3.2. Search Results on the ISI Web of Science Core Collection Platform

Search results showed 7473 research publications in SCI-EXPANDED from 1988 to 2018. Paper numbers first exceeded 100 per year in 2007 and 1000 per year in 2016. Different from the production results mentioned above, the field studies were mostly carried out in North America, Western Europe, and China, but a few in Central Asia, Central America, the Caribbean, and most parts of Africa (Figure 2C). Among the top 30 countries where the research data were collected, the highly sensitive climate threat regions based on previous trends were observed in India, Kenya, the Philippines, and Pakistan (Supplement file S2) [28]. The result indicated that lots of studies were carried out at high food production regions such as European and North American countries (20 countries in Top 30), but they were mostly under low climate change risk. In contrast, a few studies were carried out at low food production regions such as some Asian and African countries (4 countries in Top 30) where were predicted under high climate change risk [28]. Therefore, compared with the potential high climate change risk, the research advances of these Asian and African regions were far from enough.

### 3.3. Cluster Analysis Results

**Figure 3:**
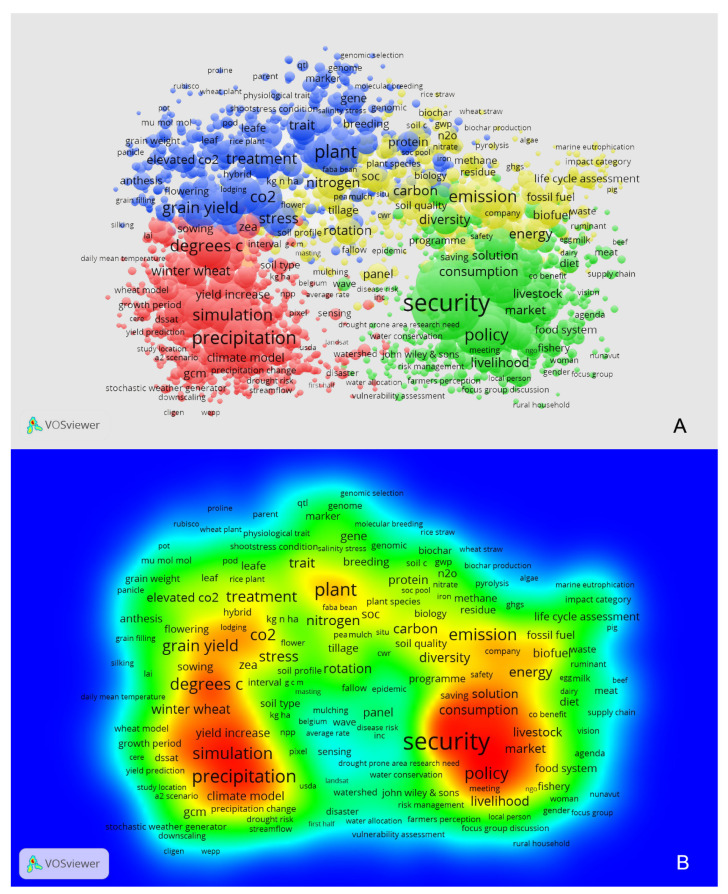
The keywords cluster analysis (**A**) and research hot-spot analysis (**B**) with the VosViewer for all scientific references of climate changes in grain productions.

In cluster analysis, the keywords with high similarities and from similar research fields were clustered into different groups and coded with different colors. In this research, the keywords were divided into four clusters that represented field and lab results (blue), model results (red), policy regulation (green), and ecology (yellow) separately (Figure 3; all results in Supplement file S1):

(1) Field and lab results (blue parts in Figure 3) were from a variety of field experimentation, such as Enclosure Chamber (EC), Open Top Chamber (OTC), Free-Air CO_2_ Enrichment (FACE), and so on. Most of these studies focused on how climate change, including atmospheric CO_2_ concentration change, could impact crop growth and productivity (macroscopic scale) or food quality or crop physiology (microscopic scale).
(2) Model results (red parts) are from those studies focused on predicting the impacts of climate change on food security at the national, regional, or even global scale. Wheat as a C_3_ model crop, which generally had a positive response to an elevated CO_2_, was selected for almost all predictions.
(3) Policy regulations (green parts), mainly discussed how climate change could affect food security through regulations of sociology, economy, and policies.
(4) Ecology (yellow parts), which focused on how climate change could affect food security through the global carbon or nitrogen cycles, including greenhouse gas emissions, tillage, and so on.

### 3.4. Results of Research Hotspot Analysis

Based on the frequency gradient of keywords from high to low, VosViewer distinguished the keywords into red, yellow, green, and blue. The red area shows the hottest topics of current research or so-called “hot research fields” (red parts in Figure 3B), and it consists of model research and policy suggestions, including models on precipitation, simulation, food security policy, and so on. The surrounding area of “hot research fields” is colored in yellow, which means hotter topics of current research, including parts of ecology considerations (emission, fuel, carbon, diversity, and so on) and field and lab results (treatment, plant, grain yield, and so on). Therefore, the most concerning keywords and questions could be summarized as “how climate change could affect food security” (model research) and “how could we deal with the impact of climate change in food security” (policy suggestions) (Figure 3).

## 4. Discussion and Sustainability Policy Suggestions

**Figure 4:**
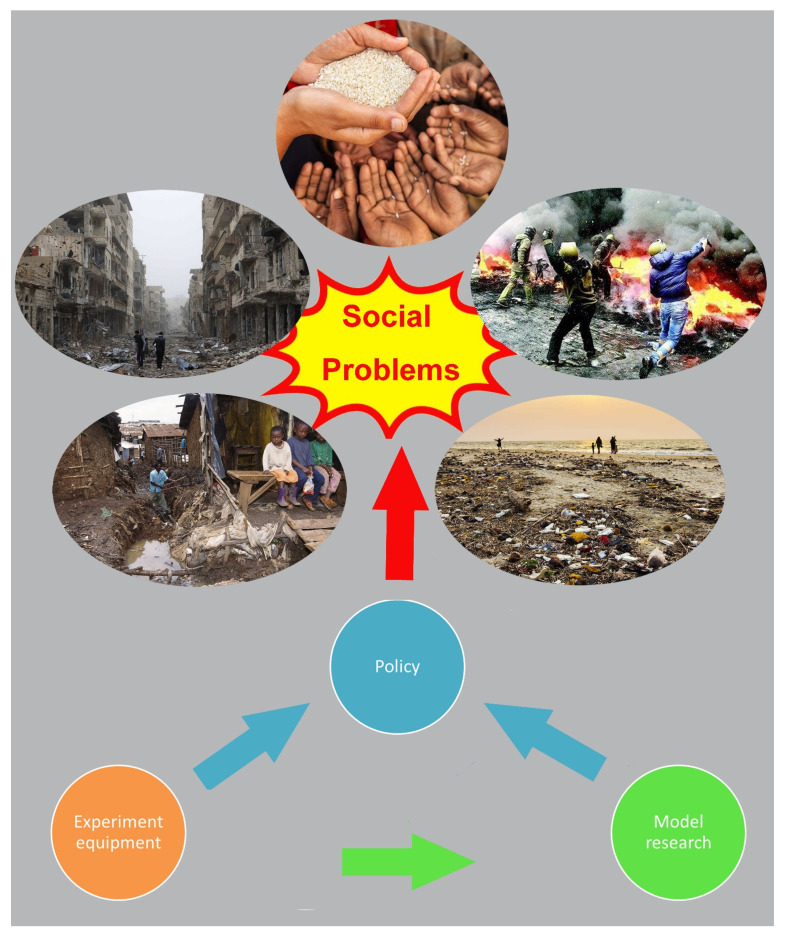
Relationships among policy suggestion, experiment equipment and model research and potential effects of climate changes on social development.

In the keyword cluster analysis, four keywords (crop model, simulation, experiments, and treatment) were related to field and model research in the top 20 most frequent words (Supplement file S1). It meant that researchers had made great efforts in both field and lab as well as model research in the past. Intense field and lab research laid a solid foundation and accelerated the rapid development of food security model studies along with providing a large amount of statistical information for relevant policy-making (Figure 4). The field and lab data were the basis for model analysis and policy-making. The model analysis results would be doubtful if the objectivity of the field and lab data were defective. What is more, these data might mislead policy-making and even induce severe social contradictions (Figure 4). Based on the extracted information from the search results, current studies were mostly carried out in European countries and the USA, a few at several Asian and African countries which had low food production and were under high climate change risk. Moreover, based on previous studies, the research data from facilities that are more authentic were still debatable. If future studies keep this trend going, the field and lab data of climate change would be more defective and then influence model analysis and policy-making.

### 4.1. Objectivity of Research Facilities

As mentioned above, the intense field and lab research produced a large amount of research data. However, the research data from which facilities were more authentic were still debatable because these data are from different facilities (e.g., EC, OTC, FACE). Moreover, these facilities were used in different experimental treatments and under various control conditions. For instance, the advantage of FACE is the open experimental condition, with minimum variation between the inside and outside conditions. However, the purchasing and operating costs for a FACE are very high whereas EC is the cheapest, and its experimental condition is easy to be regulated but shows high contrast with the external environment. The purchase and operating costs and the variation between the internal and external conditions of OTC lie between the FACE and EC. Nevertheless, there are usually discrepancies of CO_2_ concentration and temperature existing between the top and bottom locations in an OTC [29,30,31].

As a consequence of sealed experiment conditions, the compensation effect of elevated CO_2_ was overestimated in OTC or EC conditions whereas it was more authentic in FACE [32,33]. Some others reported that FACE differs from reality because CO_2_ input in FACE is stable [17,34]. Some studies also indicated that all these facilities were fundamentally the same [24,35]. In general, data from EC and OTC were higher than those from FACE in most research and the data from these chamber research studies did not follow normal distribution [36].

Actually, how environmental and climate factors could affect crop productivity is quite complicated because climate factors always change simultaneously with elevated CO_2_ concentration. It would be hard to reflect the results from EC, OTC, or FACE independently.

### 4.2. Representativeness of Field Research Site Selection

As mentioned above, some Asian and African countries which were at the low research level, had low food production and were under high climate change threat. What was worse, even in these countries, they also faced different climate change and food insecurity threats.

The per capita food production of mid-Africa is approximately one-tenth of the world average. Even worse, this region also suffers from the threat of both wars and climate change. The per capita food production of eastern Africa is a little higher than mid-Africa. The two regions are under the same climate risk: high temperature and space–time difference in precipitation. The per capita food production of western Africa was equal to that of mid-Africa before the 1980s, and then increased gradually after the mid-1980s, and has reached 25% of the world average. This region is close to the Sahara, where the weather is hot but has adequate rainfall. In contrast to other African areas, northern Africa is arider. There are limited farmlands in this region due to the presence of the Sahara. The per capita food production was higher in southern Africa than the world average before the 1980s but became lower after the mid-1990s. As it is far away from the Equator, weather in southern Africa is cool with adequate rainfall, and this area suffers little from climate change [16,37,38,39].

**Figure 5:**
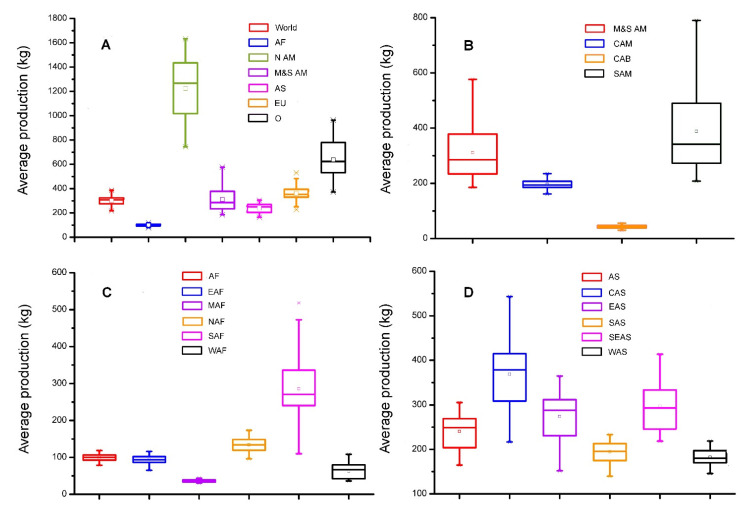
Comparisons on world average grain production over the past 55 years (1960–2014) at different continents (**A**), Mid and South America (**B**), Africa (**C**) and Asia (**D**). Abbreviations: AF, Africa; NAM, North America; M and S AM, Mid and South America; AS, Asia; EU, Europe; O, Oceania; CAM, Central America; CAB, Caribbean; SAM, South America; EAF, Eastern Africa; MAF, Mid-Africa; NAF, Northern Africa; SAF, Southern Africa; WAF, Western Africa; CAS, Central Asia; EAS, East Asia; SAS, Southern Asia; SEAS, South-east Asia; WAS, Western Asia.

Asia is one of the major grain-producing regions in the world. The production of wheat and rice is the highest in the world while maize and soybean are also widely cultivated in Asia (Figure S1 and Supplement file S2) [35,36]. However, the per capita food production of Asia is below that of the world average because of the large population (Figure 1, Figure 2D, Figure 5 and Figure S1).

The per capita food production of western Asia is quite low because of dry and drought weather, infertile farmland, and water shortage. The per capita food production of southern Asia is below that of the world average because of the large population, hot weather, and backward agricultural technology. East Asia is located in the temperate zone and is suitable for crop cultivation, so it is one of the most important agricultural, yet highly populated, regions of the world. The per capita food production of East Asia is only close to the world average because of the large population. In the context of climate change, this area is also at future risk of increased average temperature (Figure S2). Southeast Asia is located in the tropics and has abundant rainfall, so this region is one of the most important paddy production areas of the world (Figure S1). The per capita food production of this area is higher than the world average. However, countries of this region are mostly composed of islands and easy to be affected by extreme weather. The per capita food production of central Asia is higher than that of the world average with large fluctuations between years. This region is one of the most important wheat production areas of the world. However, the average temperature from 1970 to 2000 had increased by 1.5 °C, which could potentially threaten its wheat production [37,38,39].

Besides, the per capita food production in the Caribbean is much less than that of the world average (Figure 1, Figure 2D, and Figure 5). This area largely consists of tropical islands with very little farmlands and is susceptible to extreme weather [22,40].

Even though the food security in these regions is not optimistic, there are a few field studies carried out in most parts of Africa, Central America, the Caribbean, and Central Asia (Figure 2C) [28]. What was worse, Central Asia and most parts of Africa were predicted as regions highly sensitive to the climate change threat (Figure S1). A previous study projected an increase in hot nights, long and persistent heatwaves, and disaster risks that could lead to an increase by 1.5 °C or even 2.0 °C across Africa [23]. As the study region is diverse in features, the acquired field data could not be considered representative. More validated field studies in African, Asian, and Caribbean regions would help us to better understand the “effects of climate change on food security.”

### 4.3. Suggestions

Considering the above-mentioned two drawbacks, our understanding of “how climate change affects food security” was influenced to a certain extent. In future studies, researchers should pay attention to those drawbacks. Here we tried to give several suggestions.

First, experimental methodology, including equipment and data processing, should be modified or integrated to ensure comparability of the field research results. As mentioned above, because of various field research facilities and their defects, the research data from facilities that are more authentic were still debatable. Therefore, field research facilities need to be improved. Currently, there are two solutions suggested to overcome this issue. On the one hand, these facilities should be combined to take advantage of their respective unique features. On the other hand, to integrate experiment results from various equipment, new mathematical models should be developed and modified to optimize these results [41,42]. As mentioned in previous literature, FACE has the advantage of predicting how crops could respond to future climate change because of the open experimental condition. However, chambers had the advantages of identifying the mechanisms of crop response at the molecular, biochemical, and physiological scales as their experimental conditions are easy to be regulated [36]. Therefore, the choice of the experimental methodology should depend on the purpose of the research by utilizing the respective advantages and reducing the errors in the equipment. Owing to its high operating costs, we suggest that FACE should be carried out only in the regions that are highly sensitive to climate change threats in the developed countries whereas due to the reasonable cost, chamber facilities could be utilized widely in controlled experiments in most other regions. Furthermore, new modified mathematical models should be utilized to process and integrate the data from various facilities and regions. Thus, the predicted results could be verified in the facilities using the measures mentioned above.

Second, as mentioned above, there were regional differences between countries and areas in current research, which were also proved in our results [25,43]. Therefore, to gain comprehensive data, more field research should be carried out in regions of the low-income developing countries that are highly sensitive to the climate change threat (Figure 2C and Figure S2). On the one hand, such research studies would assist the developing countries to explore how to increase grain production under climate change threats and relieve grain production stress. On the other hand, the studies would also assist scientists to collect more abundant and comprehensive data to cope with future climate change. However, the research should consider the local environmental, development, economic, and other conditions. In regions highly sensitive to climate change threat, especially those in the low-income developing countries, long-term and persistent monitoring research should be carried out where there are no adequate experimental facilities whereas FACE and chamber research could be carried out at other high-income developing and developed countries. As the climate is volatile in regions highly sensitive to climate change threats, field experiments are cost-effective and could reflect how the harsh environment impacts crops directly. As the climate is mostly mild in the high-income developing and developed countries, experiments cannot reflect how extreme environments impact crops directly, so they should be carried out under regulated experimental conditions and by simulating the climate change situation.

Based on the basic data from different regions and facilities, current agricultural facilities could also be modified, upgraded, and combined with advanced technology to cope with future climate change threats [44,45]. Agricultural facilities could provide suitable crop-growing conditions internally and resist crops from external severe climate conditions; this is a mature technology to cope with climate change threats. However, there are also some challenges that should be overcome, such as efficient cooling and ventilation technologies in the tropical regions, efficient heating technologies in the polar zones, cladding materials outside facilities, and so on [46]. Similar to the various climatic and economic conditions in different countries, local conditions, such as facility costs and climatic conditions should be considered when field research is carried out.

## 5. Conclusion

Food security has been one of the hot topics of research for years. As there are large differences in the economies and states of social development, food security across countries faces different challenges under the threat of climate change. However, as shown in this article, too many studies were carried out in the regions under low food insecurity, only a few in regions with high food insecurity. In the meantime, large-scale research data were collected from different facilities, or even under various experimental treatments and control conditions. Therefore, the objectivity of field research facilities and representativeness of field research site selection is still debatable. Therefore, we wish to make the following proposals: First, different kinds of facilities should be combined to take advantage of their respective unique features to reflect influences under various climatic conditions. Second, to integrate experiment results from various equipment, new mathematical models should be developed and modified to optimize these results. Third, to gain comprehensive data, more field research should be carried out in regions of low-income developing countries that are highly sensitive to climate change threats. To frame the rules as objectively as possible, abundant and suitable data are required. The data should be not only of a large size but also of a representative nature. This large requirement cannot be met by individual institutes or countries. The whole world should be united to join in this project actively and cope with the potential climate change threat positively.

## Data Availability

The datasets generated for this study are available on request to the corresponding author.

## Supplementary Materials

The following are available online at https://www.mdpi.com/article/10.3390/foods10102342/s1, Figure S1: Global cultivation areas (**A**) and yields (**B**) of maize, rice, wheat and soybean, Figure S2: Changes and frequencies of abnormal surface temperature per 10 years from 1970 to 2000, Supplement file S1: Keywords cluster analysis in Vosviewer, Supplement file S2: Search results of 7473 research publications from SCI-EXPANDED, Supplement file S3: The global and regional food crop production data
