# Supplementary material for: Is Current Research on How Climate Change Impacts Global Food Security Really Objective?
Source: Foods. 2021 Sep 30;10(10):2342. doi: 10.3390/foods10102342 (PMC8535570; doi:10.3390/foods10102342)
Supplement: Supplementary file 1 [file foods-10-02342-s001.zip › Supplement figures.pdf]

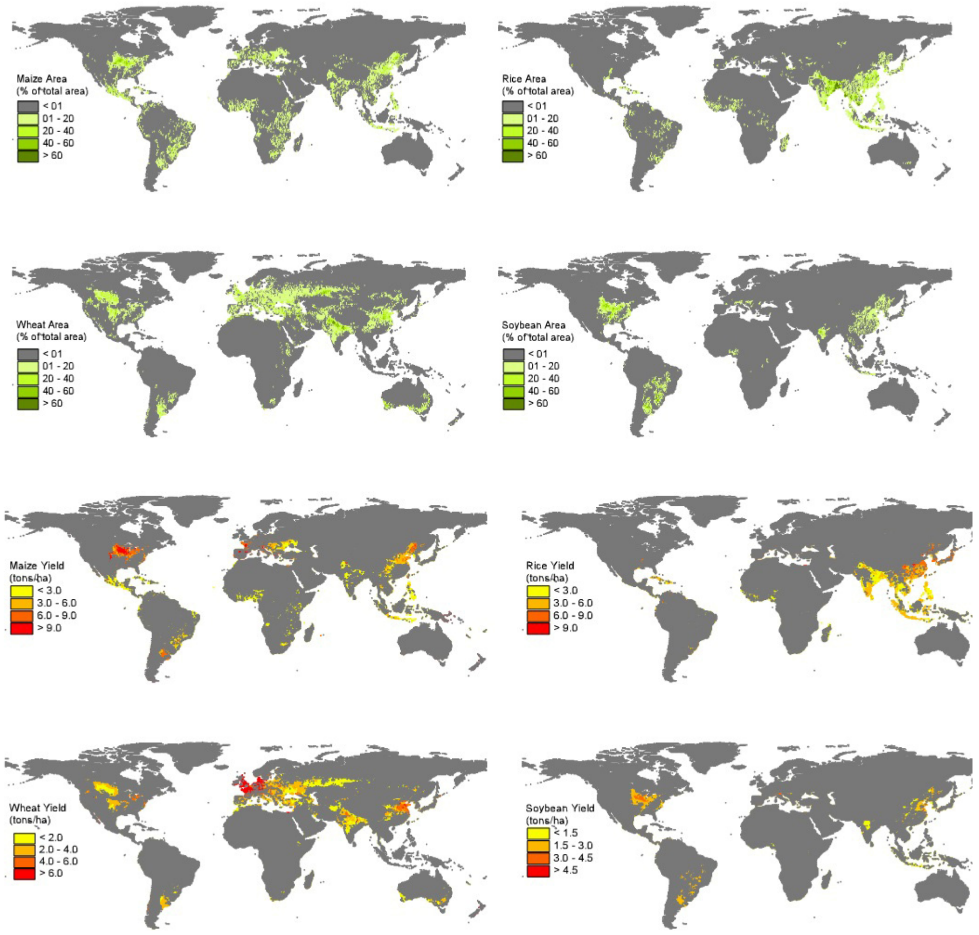

Figure S1. Global cultivation areas (A) and yields (B) of maize, rice, wheat and soybean [28].

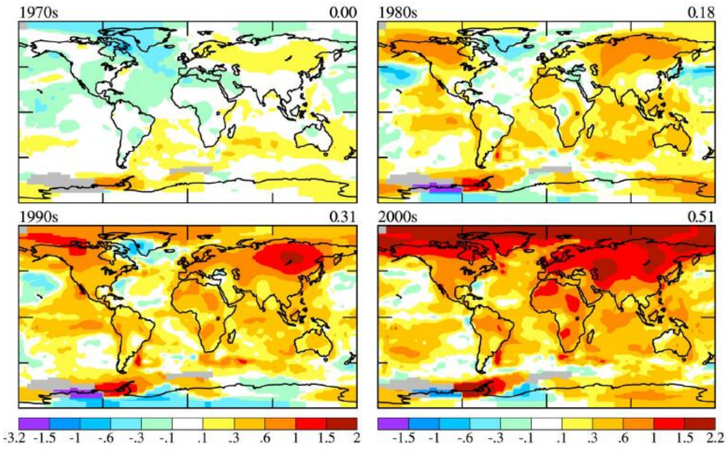

Figure S2. Changes and frequencies of abnormal surface temperature per 10 years from 1970 to 2000 [47].

47 Monfreda, C.; Ramankutty, N.; Foley, J., A. Farming the planet: 2. Geographic distribution of crop areas, yields, physiological types, and net primary production in the year 2000. *Glob. Biogeochem. Cycles* **2008**, *22*, 1–19. GB1022, doi:10.1029/2007GB002947.
